# Quantum crystallography†

**DOI:** 10.1039/c6sc05504d

**Published:** 2017-03-27

**Authors:** Simon Grabowsky, Alessandro Genoni, Hans-Beat Bürgi

**Affiliations:** a Universität Bremen, Fachbereich 2 – Biologie/Chemie, Institut für Anorganische Chemie und Kristallographie Leobener Str. NW2 28359 Bremen Germany simon.grabowsky@uni-bremen.de; b CNRS, Laboratoire SRSMC, UMR 7565 Vandoeuvre-lès-Nancy F-54506 France; c Université de Lorraine, Laboratoire SRSMC, UMR 7565 Vandoeuvre-lès-Nancy F-54506 France Alessandro.Genoni@univ-lorraine.fr; d Universität Bern, Departement für Chemie und Biochemie Freiestr. 3 CH-3012 Bern Switzerland hans-beat.buergi@krist.unibe.ch; e Universität Zürich, Institut für Chemie Winterthurerstrasse 190 CH-8057 Zürich Switzerland

## Abstract

Approximate wavefunctions can be improved by constraining them to reproduce observations derived from diffraction and scattering experiments. Conversely, charge density models, incorporating electron-density distributions, atomic positions and atomic motion, can be improved by supplementing diffraction experiments with quantum chemically calculated, tailor-made electron densities (form factors). In both cases quantum chemistry and diffraction/scattering experiments are combined into a single, integrated tool. The development of quantum crystallographic research is reviewed. Some results obtained by quantum crystallography illustrate the potential and limitations of this field.

## Abstract 2

Quantum crystallography combines quantum chemistry and experimental diffraction or scattering to provide both enhanced wavefunctions and charge densities.

## Graphical abstract

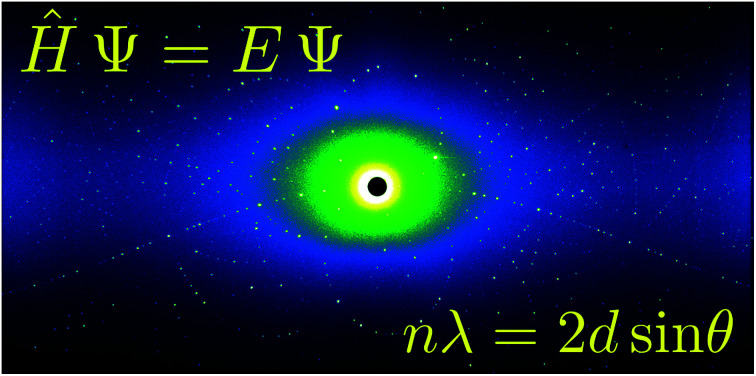

## 1. Introduction

Quantum chemistry methods and crystal structure determination are highly developed research tools, indispensable in today's organic, inorganic and physical chemistry. These tools are usually employed separately. Diffraction and scattering experiments provide structures at the atomic scale, while the techniques of quantum chemistry provide wavefunctions and properties derived from them. In this contribution we review different efforts towards combining tools of these two fields into integrated quantum crystallographic strategies.

**Fig. 1:**
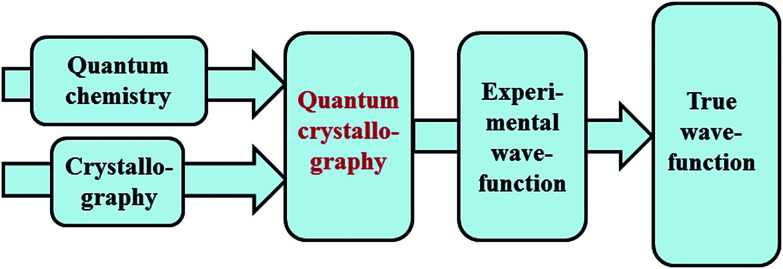
Quantum crystallography, first aspect: crystallographic data are integrated into quantum chemical calculations to enhance the information content of the wavefunction. The resulting, so-called “experimental wavefunction” represents an improved approximation to the true wavefunction.

**Fig. 2:**
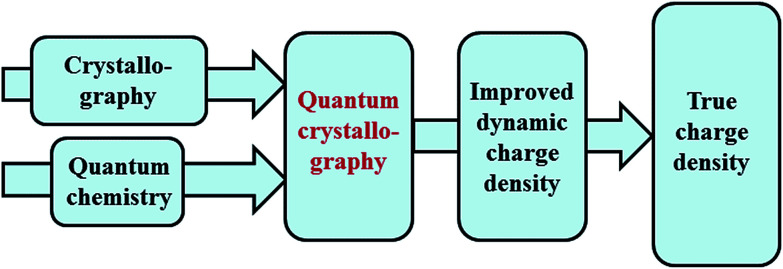
Quantum crystallography, second aspect: quantum chemical calculations are integrated into crystal structure determination to improve the dynamic charge density, *i.e.* the thermally smeared electron and nuclear densities.

The term quantum crystallography was first introduced by Massa, Huang and Karle in 1995 for methods that exploit “crystallographic information to enhance quantum mechanical calculations and the information derived from them”.^1^ The basic idea is to compensate shortcomings and limitations of quantum mechanical models, *e.g.* incomplete consideration of electron correlation, with experimental data that are not suffering from the same limitations (Fig. 1). In 1999 the same authors also suggested the converse possibility: “… quantum mechanics … can greatly enhance the information available from a crystallographic experiment” (Fig. 2).^2^

Mutually subsidiary combinations of quantum chemistry and X-ray structure determination suggest themselves.^3^ Quantum chemical models are usually based on some approximations of the true wavefunction. X-ray structure determination aims at the true charge density, which is related to the square of the true wavefunction, but is affected to a smaller or larger extent by vibrational motion and experimental errors.

**Fig. 3:**
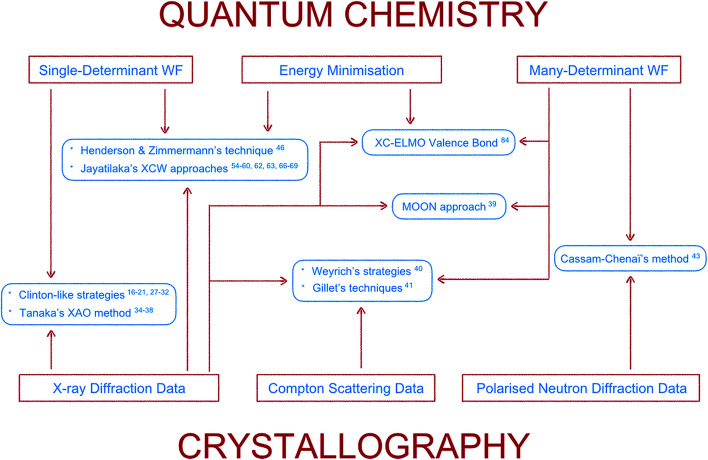
Scheme summarizing the features (framed in red) of the main methods (framed in light blue) according to the first definition of quantum crystallography. The lower the position of the method is in the scheme, the lower is the quantum chemistry contribution in it. Each family of techniques is associated with the corresponding bibliographical references in the paper.

Here the topic of quantum crystallography is presented in two parts. The first part summarizes the development of increasingly sophisticated methods to combine information from quantum chemical calculations with diffraction and other experimental data (see Fig. 3 for a summarizing scheme). The second part describes ways of improving structural models obtained from diffraction experiments by combining them in a self-consistent way with information from quantum chemical calculations.

## 2. Quantum crystallography, first definition: enhancing quantum chemical calculations with experimental information

### 2.1 Pioneering “experimental” wavefunction techniques

All modern quantum crystallography techniques according to the first definition originate from the pioneering “experimental” wavefunction strategies originally proposed in the 1960s by Mukherji and Karplus.^4^ They perturbed unconstrained Hartree–Fock molecular orbitals until they produced satisfactory agreement with experimental dipole moments or electric field gradients at a minimal increase in the energy of the system. Agreement improved not only for the constraining experimental values, but also for other properties, such as diamagnetic and paramagnetic susceptibilities. Rasiel and Whitman^5^ introduced experimental dipole moment constraints‡‡In all reviewed methods according to the first definition, the authors refer to the use of external parameters as “constraints”. Hence, we will use this term throughout Chapter 2, although we believe that, in a crystallographic sense, the term “restraints” would be more appropriate. into the variational minimization of the energy with Lagrange multipliers. Byers Brown and Chong^6^ proposed to implicitly introduce the experimental constraints by adding proper quantum mechanical operators *Ô*_*i*_ (multiplied by a Lagrange multiplier *λ*_*i*_) to the starting and unconstrained Hamiltonian *Ĥ*:1
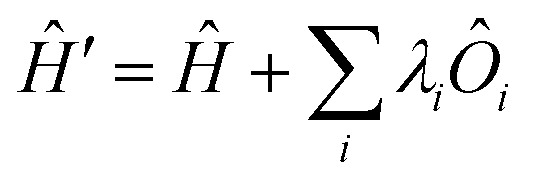


The modified Schrödinger equation can be solved variationally as shown by Fraga and Birss.^7^

In 1969 Clinton *et al.* published a series of groundbreaking ideas that may be considered the direct precursors of today's quantum crystallography methods.^8–11^ Although X-ray diffraction data were not considered explicitly, the proposed theoretical framework forms the basis for the methods developed in the 1970s and 1980s that combine quantum mechanical calculations with crystallographic data. In the first paper of the series,^8^ the authors presented a semi-empirical strategy to determine one-electron density matrices§§The one-electron density matrix *γ*(***x***;***x*′**) for a pure state of an N-electron system is related to the global wavefunction *ψ*(***x***_1_,***x***_2_,…,***x***_*N*_) for the same pure state of the system through the following relation: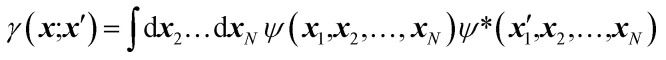 where ***x*** and ***x*′** are two sets of independent space and spin coordinates (*i.e.*, ***x*** = {***r***,*s*} and ***x*′** = {***r*′**,*s*′}). Since the numerical value of *γ* changes according to the value of ***x*** and ***x*′**, *γ*(***x***;***x*′**) can be seen as a matrix of infinite dimensions. It intrinsically contains all the information about the one-electron properties of a system and, if integrated over the continuous spin coordinates, reduces to the spinless one-electron density matrix:
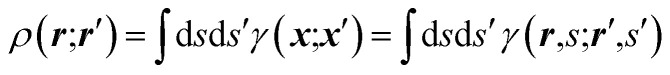
whose diagonal part corresponds to the electron density (*ρ*(***r***;***r***) = *ρ*(***r***)). Working with a finite set of *M* basis functions {*χ*_*μ*_} (*i.e.* the usual basis-sets of quantum chemistry), the spinless one-electron density matrix can be expressed in terms of the finite matrix ***P***:
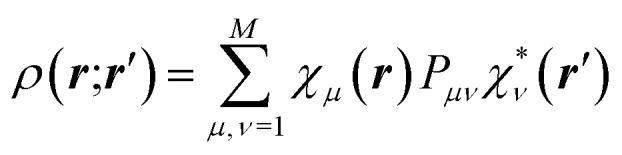
 of small diatomic molecules. They had both theoretical and practical reasons for focusing on one-electron density matrices rather than the more usual wavefunctions.^8,9^ To accomplish their task, they used theoretical constraints, such as the Hellmann–Feynman and virial theorems in combination with experimental constraints, *e.g.* potential energy curves obtained from spectroscopic and scattering measurements. However, the density matrices initially obtained by Clinton *et al.* did not conform with the essential quantum mechanical requirement that each density matrix must correspond to an antisymmetric wavefunction (N-representability).^12–14^ For single Slater determinant wavefunctions, this drawback can be overcome by imposing the idempotency condition (*i.e.*, ***P***^2^ = ***P***) and the normalization to the number of electrons (Tr***P*** = *N*) on the one-electron density matrices.^9^ This goal was achieved by combining the original, iterative McWeeny density matrix purification formula^13^

2 ***P***_*n*+1_ = 3***P***_*n*_^2^ − 2***P***_*n*_^3^

with (i) the Hellmann–Feynman and virial theorems,^9^ (ii) Parr's integrated Hellmann–Feynman theorem,^10^ (iii) local energy constraints^11^ and, later, (iv) cusp conditions.^15^ This gave rise to another iterative procedure in which the density matrix at each iteration is obtained according to the following equation:3
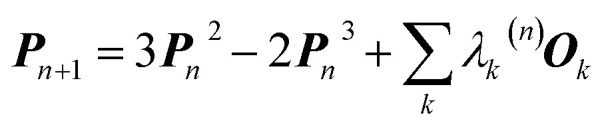
where ***P***_*n*+1_ and ***P***_*n*_ represent the one-electron density matrix in the adopted finite set of basis functions at the *n* + 1 and *n* iterations; ***O***_*k*_ is the matrix representation of the operator *Ô*_*k*_ associated with the property *k* in the same finite basis set as the density matrix. The external multipliers {*λ*_*k*_^(*n*)^} are determined in a separate calculation by solving an auxiliary set of linear inhomogeneous equations¶¶
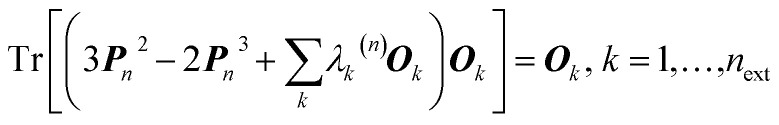
. The unknowns for the resulting system of inhomogeneous equations are the external multipliers {*λ*_*k*_^(*n*)^}. that constrain the density matrix to reproduce the values {*o*_*k*_} of the properties considered.

### 2.2 The first quantum crystallographic methods: the original Clinton & Massa approach and its later developments

An X-ray diffraction experiment provides a large number of structure factor amplitudes, observables from which the electron density in the crystal can be determined (after recovering their phases). Recognizing this fact Clinton and Massa attempted for the first time to extract a one-electron density matrix from X-ray diffraction data.^16^ Their 1972 technique may be considered as the first quantum crystallography investigation. It differs from the strategy proposed by Clinton and coworkers in 1969 (ref. 9) only by using structure factors as external constraints and basically consists in iteratively solving the following matrix equation:4
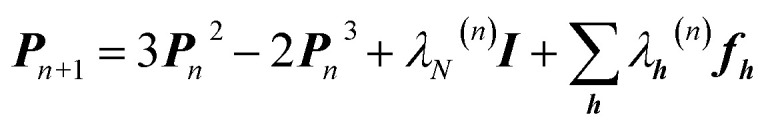
where ***P***_*n*+1_ and ***P***_*n*_ have the same meaning as in eqn (3), ***I*** is the identity matrix, ***f***_***h***_ is the matrix of the Fourier transforms of the basis functions products, ***h*** is a triad of Miller indices labeling the reflection considered, *λ*_*N*_^(*n*)^ and *λ*_***h***_^(*n*)^ are external multipliers determined at each iteration from the auxiliary set of equations that constrain the density matrix to be normalized to the correct number of electrons and to reproduce the given set of structure factors.

Initially Clinton, Massa and others studied very small model systems at rest (mainly atoms and the hydrogen molecule) with structure factors calculated from near-exact wavefunctions.^16–21^ In 1973 they generalized their algorithm to take into account real experimental data with their errors (but did not test it with actual data).^17^ Because of the experimental errors, the structure factor constraints cannot be satisfied exactly but only in a least-square sense.^17^ Frishberg^22^ compared in detail the values of some physical properties for the beryllium atom predicted by the near Hartree–Fock wavefunction of Clementi^23^ on one hand and by the X-ray fitted^19^ and Best Density Matrix^24,25^ (BDM) wavefunctions on the other hand. In most cases the one-electron properties obtained with the quantum crystallography strategy agreed best with the results calculated from the highly accurate and correlated wavefunction^26^ used as reference. As far as momentum-related properties are concerned, the predictions based on the X-ray fitted wavefunction were always superior to the near Hartree–Fock ones. However, the BDM technique made the best predictions.

The investigations summarized so far established the theoretical basis for combining quantum chemical calculations with experimental X-ray diffraction data as external constraints, showed its potential and its limitations, but used only calculated X-ray structure factors (usually for atoms at rest). The first density matrix determined from experimental X-ray structure factors was that of beryllium in 1985.^27^ In a later study by Aleksandrov *et al.*^28^ one-electron density matrices for silicon and diamond were successfully fitted to experimental structure factors. Electronic kinetic energies and Compton profiles derived from them were in good agreement with corresponding experiments.

Over the years several modifications and improvements of the Clinton & Massa “experimental” density matrix method were proposed. Massa and coworkers extended the original approach (i) to the case of open-shell systems^19^ and (ii) to extended systems using Bloch and Wannier functions.^29^ The latter extension takes into account the interaction among unit cells and allows to study insulators, semiconductors and metals, at least in principle. The most important step forward for this family of techniques is probably the one proposed by Pecora.^30^ He devised a simplified steepest-descent algorithm to determine “experimental” idempotent density matrices. It is mainly based on the gradient of the average squared error between calculated and experimental structure factors amplitudes (*i.e.*, the well-known *χ*^2^ statistic). With his approach, he provided one of the earliest density matrices obtained from real experimental data, positron annihilation data for a Cu–Ge alloy in his case. Pecora's algorithm was further improved by Howard *et al.*^31^ The resulting powerful simulated annealing strategy allowed for the first time to study molecules as large as methylamine and formamide, for which theoretical and experimental X-ray diffraction data were used as external constraints, respectively. In a follow-up investigation Snyder and Stevens^32^ determined the density matrix for the azide ion in potassium azide from experimental structure factors. They found that the electron density associated with the fitted density matrix shows some features that are common both to the multipole model (see Section 3.2) and to theoretical charge distributions used for comparison.

Among the pioneering quantum crystallography methods, the strategy proposed by Figgis and collaborators has to be mentioned.^33^ They extracted populations of valence atomic orbitals from X-ray or polarized neutron diffraction data, *e.g.* in coordination complexes such as the *trans*-tetraamminedinitronickel(ii) [Ni(ND_3_)_4_(NO_2_)_2_] compound and the CoCl_4_^2−^ anion in Cs_3_CoCl_5_. The X-ray Atomic Orbital (XAO) method developed by Tanaka and coworkers also belongs to this family of approaches.^34–38^ It determines atomic orbitals on transition metal ions by minimizing the usual statistical agreement between experimental and theoretical structure factors subject to orbital orthonormality and symmetry constraints. Although the strategy initially aimed at modeling the crystal-field influence on the d atomic orbitals,^34,35^ it was later extended to also treat s, p and f functions.^36–38^

### 2.3 Beyond the single Slater determinant approximation

All the methods described so far suffer from an intrinsic limitation: since they require the one-electron density matrices to be idempotent, these matrices are necessarily associated with single Slater determinant wavefunctions. The MOON (Molecular Orbitals Occupation Numbers) method^39^ tries to overcome this problem by re-determining the occupation numbers of pre-computed occupied and virtual molecular orbitals, such that the agreement with the X-ray diffraction data is maximized. The current version of the method offers two options. In the first the optimized occupation numbers of the molecular orbitals are constrained to be between 0 and 2, which implies that one-electron density matrices are N-representable. In the other option, the upper limit on the occupation numbers is dropped. This introduces more flexibility in the fitting process, but may lead to results violating the Pauli principle. In both options the coefficients of the pre-determined molecular orbitals are not optimized during the fitting procedure; the technique thus scales linearly with the size of the basis set. However, the necessity to pre-compute the molecular orbitals renders the application of MOON to very large molecules quite difficult.

More advanced methods to overcome the single-determinant limitation replace the basic idempotency constraint with a more general N-representability condition that requires the eigenvalues for the one-electron density matrix to lie between 0 and 1.^14^ Weyrich and collaborators^40^ introduced this condition into their pioneering methods for the reconstruction of the complete one-electron density matrix (*i.e.* including diagonal and non-diagonal parts) from a combination of X-ray diffraction and inelastic Compton scattering data. Gillet and coworkers followed the same philosophy in more recent studies.^41^ Weyrich's technique was extended by Nicholson *et al.*^42^ to obtain Dyson orbitals from electron momentum spectroscopic data. The strategy devised by Cassam-Chenaï is included here in the same group of techniques;^43^ a small number of wavefunctions (obtained through preliminary *ab initio* calculations) are used to expand the ensemble N-representable one-electron density matrices of the systems investigated. The expansion coefficients are determined through a fit to the experimental measurements. Using this idea Cassam-Chenaï *et al.*^44^ determined the spin density of the CoCl_4_^2−^ anion in the Cs_3_CoCl_5_ crystal from polarized neutron diffraction data.

### 2.4 Criteria to select the best wavefunction

The theoretical developments and practical strategies outlined above represent an important basis for combining quantum mechanical calculations with experimental data. However, as Gilbert has pointed out, they suffer from a further ambiguity.^45^ There is an infinite number of N-representable one-electron density matrices (differing only in their off-diagonal parts) and, consequently, an infinite number of wavefunctions that are compatible with a given electron distribution. Therefore, even under strict N-representability constraints, simple fitting to density data may lead to an inappropriate density matrix or wavefunction. Henderson & Zimmermann^46^ proposed that, among all single Slater determinants that reproduce a given electron density, the best one is the one that minimizes the Hartree–Fock energy functional. They introduced this criterion into the 1972 approach of Clinton & Massa.^16^ Their algorithm modifies in a quasi-continuous way a starting Hartree–Fock wavefunction until the desired X-ray constrained solution is obtained at a minimum penalty in energy.^46^ A similar philosophy was followed by Kryachko and coworkers.^47^ Their original method of local-scaling transformations^48^ was exploited to modify an initial wavefunction (the promolecule wavefunction) until it reproduces the electron density given by the experimental X-ray diffraction data. In this approach the energetic criterion of Henderson & Zimmermann was not considered explicitly.

Levy & Goldstein^49^ and later Gritsenko & Zhidomirov^50^ proposed an alternative to the Henderson & Zimmermann criterion. They select the single Slater determinant with minimal kinetic energy among the ones compatible with a given charge distribution. Zhao, Morrison and Parr^51^ used this idea in the early 1990s to extract Kohn–Sham orbitals from electron density data generated by *ab initio* computations. Orbitals obtained with this philosophy are almost identical with the corresponding Hartree–Fock ones. For this reason the authors proposed (i) that the Kohn–Sham single Slater determinants obtained by kinetic-energy minimization could represent physically meaningful wavefunctions and (ii) that their strategies could provide an alternative way towards proper wavefunctions from experimental data. Other techniques to extract wavefunctions and crucial DFT quantities from the electron density have previously been introduced by Nyden & Parr^52^ and by March and coworkers.^53^ Unfortunately, their strategies have never been tested with real experimental data as external constraints.

### 2.5 Jayatilaka's X-ray constrained wavefunction fitting: the original strategy and recent advances

Nowadays, the most reliable and practical implementation of the Henderson & Zimmerman idea is the X-ray constrained wavefunction (XCW) fitting approach devised by Jayatilaka.^54–60^ The method is currently implemented for molecular crystals. It assumes identical, space group-symmetry related, non-interacting molecules. The unit-cell electron density of the molecular crystal is expressed in terms of the electronic wavefunction *ψ* of a reference molecule and its symmetry related copies. To guarantee that the unit-cell electron density of the fictitious non-interacting molecular crystal is identical to the unit-cell electron distribution of the corresponding real interacting system, the Jayatilaka method determines the wavefunction *ψ* of the reference molecular unit that minimizes a new functional given by the sum of the energy of the molecule and of an additional term measuring the statistical agreement between the experimental and the calculated structure factor amplitudes:

5 *J*[*ψ*] = 〈*ψ*|*Ĥ*|*ψ*〉 + *λ*(*χ*^2^[*ψ*] − Δ)

where *λ* is an external multiplier manually adjusted during the “fitting procedure” until achieving the desired statistical agreement Δ between the theoretical and the experimental structure factors (typically fixed to 1.0). *χ*^2^ is the statistical agreement between the calculated and the experimental structure factor amplitudes:6
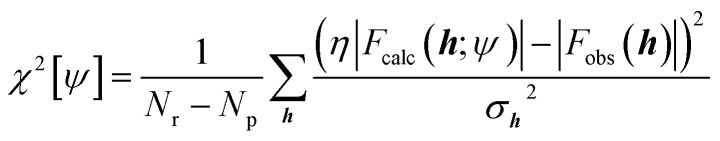
with *N*_r_ the number of measured X-ray diffraction data, *N*_p_ the number of adjustable parameters, ***h*** the triads of Miller indices labeling the reflections, *σ*_***h***_ the standard uncertainty of |*F*_obs_(***h***)|, and *η* a scale factor properly determined to minimize *χ*^2^.

From a theoretical point of view, the XCW method is fully supported by the Levy–Lieb theorem,^61^ according to which the exact wavefunction of a system not only provides its electron density, but also minimizes the sum of the kinetic and the electron–electron repulsion energies. For the same reason, the Henderson & Zimmerman criterion introduced in the previous subsection is to be preferred to the one based on the minimization of only the kinetic energy.

The XCW fitting strategy was originally developed in the framework of the restricted Hartree–Fock approach, but was later extended to the unrestricted case,^62^ to the relativistic scalar second-order Douglas–Kroll–Hess^62^ and Infinite-Order Two Component (IOTC) methods.^63^ It is also available in conjunction with the Kohn–Sham formalism.^60^ Recently the Jayatilaka method was coupled with a technique developed by Stoll *et al.*^64^ to determine Extremely Localized Molecular Orbitals (ELMOs).^65^ The resulting X-ray constrained ELMO method^66–69^ allows the extraction of X-ray constrained molecular orbitals strictly localized on small molecular fragments, such as atoms, bonds or functional groups.

### 2.6 Applications of the XCW fitting technique

**Fig. 4:**
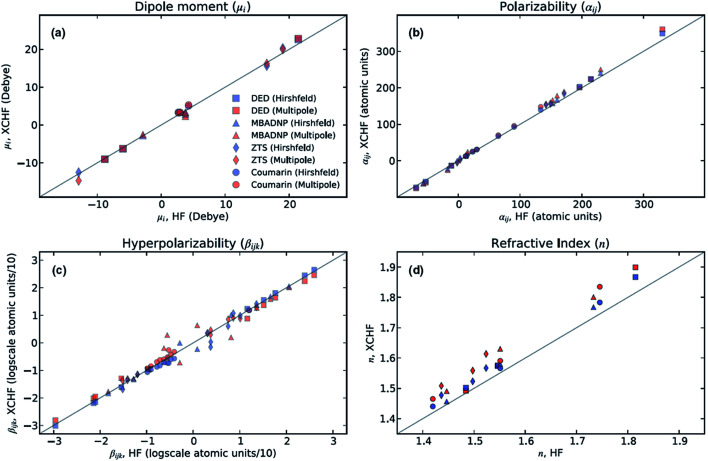
Comparison of optical properties calculated at the Hartree–Fock level with those calculated from XCWs. Two slightly different geometries and sets of atomic displacement parameters were used for the XCW fit, one based on a multipole refinement, the other on a Hirshfeld atom refinement model (see Section 3.3). Reprinted from ref. 72 with the permission of AIP Publishing.

The XCW fitting method does not only provide reliable electron density distributions. From XCWs, physical properties of materials,^70–73^ such as non-linear optical properties, can be determined. These properties cannot be obtained from experimental electron densities since the crucial many-electron contributions would be missing.^70^ In 2013, Hickstein *et al.*^72^ exploited the Jayatilaka technique to compute dipole moments, molecular polarizabilities, hyper-polarizabilities and refractive indices for four compounds: coumarin (1-benzopropan-2-one), DED ({4-[bis(diethylamino)-methylium]phenyl}dicyanomethanide), MBADNP (3,5-dinitro-2-[1-phenyl-ethyl]aminopyridine) and ZTS (zinc [tris]thiourea sulphate), all of them displaying interesting non-linear optical properties. The authors claim^72^ that the properties calculated from XCWs agree with those calculated at the Hartree–Fock level with an average statistical deviation of 20% from each other (see Fig. 4), which is comparable to the statistical deviation between Hartree–Fock and experimental values for molecular polarizabilities.

In a follow-up study, Cole and Hickstein^73^ analyzed ZTS in more detail. They observed that the refractive index derived from the static linear polarizability which in turn was calculated from the XCW (atomic positions and displacement parameters from the multipole model) agrees quite well with the experimental value unlike the corresponding gas phase value calculated *ab initio*. The authors concluded that XCW fitting implicitly accounts for the solid-state effects present in the condensed phase. The same investigation also highlighted the fact that values of optical properties obtained from a multipole model differ from the ones resulting from *ab initio* computations and XCW fitting by up to two orders of magnitudes. This confirms the inadequacy of computing properties depending on two-electron contributions from traditional charge density models (see Section 3.2) and, consequently, the necessity of resorting to X-ray constrained wavefunctions in all those cases.

**Fig. 5:**
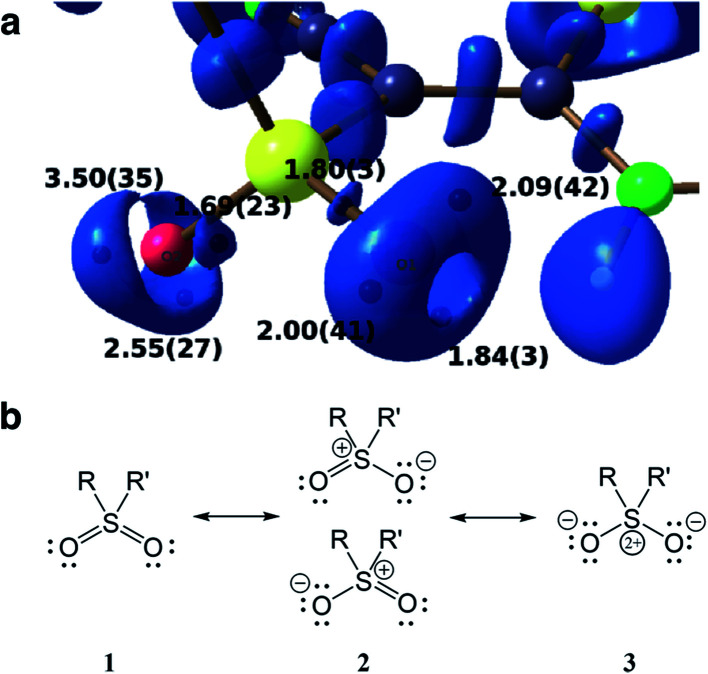
(a) Isosurface representation of the X-ray constrained ELI for a sulfonyl group (ELI = 1.445 around the S–O bond attractors and ELI = 1.550 around the lone-pair attractors) with ELI valence basin electron populations (in e, with associated standard deviations). Black spheres below the transparent ELI isosurfaces indicate the positions of the basin attractors. Each basin attractor shown can be interpreted as representing a lone pair. The oxygen atom on the right is involved in an intramolecular hydrogen bond which induces asymmetry into the electronic system of the sulfonyl group. Reproduced from ref. 80 with permission of John/Wiley & Sons, Inc. (b) Possible Lewis resonance structures of a sulfonyl group.

Another advantage of the XCW approach is its capability to include chemically important effects (*e.g.*, electron correlation and crystal effects) that can otherwise only be accounted for with a considerably larger computational effort. These can be studied by exploiting several functions and descriptors, such as the Electron Localization Function (ELF),^74^ the Electron Localizability Indicator (ELI)^75^ and bond indices,^76^ which were extracted from XCWs.^77–80^ The crystal structure of a vinyl sulfone (1-cyano-1-phenylsulfonyl-2-methylthio-2-methylaminoethylene) serves as an example.^80^ An ELI isosurface plot of the sulfonyl moiety is shown in Fig. 5a, the most important Lewis resonance structures for the sulfonyl group are depicted in Fig. 5b. In closed-shell systems the isosurfaces embrace regions of maximum localization of electron pairs and can hence be related to bonding and non-bonding valence effects. A topological analysis of the ELI provides basins for electron pairs with corresponding attractors (black spheres under the surface in Fig. 5a). Each attractor can be interpreted as representing a bonding or lone electron pair. For the oxygen atoms there are two and three attractors and thus two and three lone pairs corresponding to one double and one single bond, respectively. This points towards an asymmetric Lewis description with an increased contribution of resonance form **2** (top). The asymmetry is induced by a strong intramolecular resonance assisted hydrogen bond of one of the oxygen atoms with a neighboring amine group. Interestingly, this asymmetry in the topology is not reproduced in a Hartree–Fock calculation where two lone pairs per oxygen atom are found,^80^ but is reproduced in a highly correlated CCSD (coupled-cluster with single and double excitations) calculation (unpublished results). It is known that ELI is particularly sensitive to electron correlation. The extraction of electron correlation from single-determinant XCWs will be discussed below in more detail (Sections 2.7 and 2.8).

### 2.7 X-ray constrained wavefunction fitting: problems and outlooks

At present, XCW fitting appears to be the most promising and reliable method among the various “experimental” wavefunction strategies. However, the results of the XCW fitting depend on the quality of positional and thermal parameters and the model through which these are obtained (*i.e.*, Independent Atom Model (IAM), multipole model, Hirshfeld Atom Refinement (HAR), *etc.*, see Chapter 3). In the investigation discussed in Section 2.6, Hickstein and coworkers^72^ observed that the agreement between the results from XCW and fully *ab initio* computations improves somewhat when the XCW fitting is based on atomic positions and anisotropic displacement parameters (ADPs) resulting from a Hirshfeld atom refinement rather than from a multipole refinement (Fig. 4). In order to minimize the bias on the constrained wavefunction due to inadequate ADPs, the latter could be adapted periodically during the fitting process in an iterative version of the technique called X-ray wavefunction refinement (see Chapter 4). For a given set of ADPs the XCW will also differ depending on the way the ADPs are used in order to smear the static electron density (Stewart,^81^ Coppens^82^ and Tanaka^34^). The use of the latter three in the XCW fitting is compared in ref. 56.

There are other open questions: (i) the applications discussed in chapter 2.6 lead to the question to which extent the Jayatilaka-type approaches capture crystal field effects, electron correlation and relativistic effects on the electron density? Two recent papers by Bučinský *et al.*^63^ and by Genoni *et al.*^83^ deal with relativistic and electron correlation problems, respectively. They present perspectives for the development of new effective core potentials and density functionals from experimental X-ray diffraction data. (ii) Can the XCW approach, which is currently limited to a single determinant wavefunction *ansatz*, be extended to multi-determinant wavefunctions? Such an extension has recently been proposed^84^ in an attempt to interpret the experimentally observed suppression of aromaticity in a bis-carbonyl[14]annulene (BCA) at high-pressure.^85^ The X-ray constrained wavefunction of BCA is written as a linear combination of two pre-computed ELMO Slater determinants, which correspond to the two resonance structures of BCA and are kept frozen during the XCW fitting. The relative weights of the two determinants are fitted to the experimental structure factors (XC-ELMO valence bond approach). Further developments of the technique are currently in progress, by allowing the optimization of the ELMO Slater determinants during the computations. (iii) What is the meaning of the multiplier *λ* (see eqn (5)) and is there a clear and reliable criterion for choosing its value and thus for stopping the “fitting” procedure? Although different ideas have been proposed,^55,56,67,70,72^ a satisfactory solution has yet to be presented.

### 2.8 Tampering with quantum chemistry?

From a quantum theoretical point of view, one might argue that the quantum crystallographic approaches described in this chapter tamper with clearly defined, albeit approximate, quantum mechanical models. The wavefunctions, one-electron density matrices and energies resulting from quantum crystallographic procedures are no longer solutions of the model Hamiltonian operator chosen for the quantum calculations, but are modified by the experimental data. There seems to be no rigorous mathematical proof based on the principles of quantum theory showing that this modification also represents an improved description of the system. The improvement can only be tested by comparing the modified description with the results of the best possible calculations or with experimental results not used as constraints. This has been done for optical properties calculated from XCWs^72,73^ (see Section 2.6). The XCW single Slater determinant produces better agreement with experimental data than the simple Hartree–Fock single Slater determinant.^73^ In order to test whether the Jayatilaka approach captures effects of electron correlation on the electron density, an XCW has been fitted to structure factors obtained from (highly correlated) coupled cluster computations.^83^ The comparison between the resulting electron distribution and the (theoretical) correlated one showed that electron correlation effects are partially captured if large values of the external multiplier *λ* (see eqn (5)) are used and if mainly low-order reflections, which contain most of the information about electron correlation, are used as external constraints.^83^ These studies strongly indicate that XCW fitting strategies introduce information into the “experimental” wavefunction that is missing in the unconstrained wavefunction *ansatz*.

### 2.9 Towards enhancing models of nuclear motion with experimental information

A comprehensive model of a crystal's charge density encompasses not only a description of the electron density, but also of nuclear motion. With a good quantum mechanical model and assuming the Born–Oppenheimer approximation it is possible to calculate not only the static equilibrium electron density, but also the crystal vibrations, *i.e.* the phonon dispersion spectrum of the crystal, which can be computed by diagonalizing the dynamical matrix. From the crystal vibrations, the mean square atomic displacement parameters (ADPs) are obtained. Such calculations can become extremely intricate depending on the density of *k* points used to sample the Brillouin zone and to model the 3*nN* vibrations for a crystal containing *N* unit cells with *n* atoms each.

**Fig. 6:**
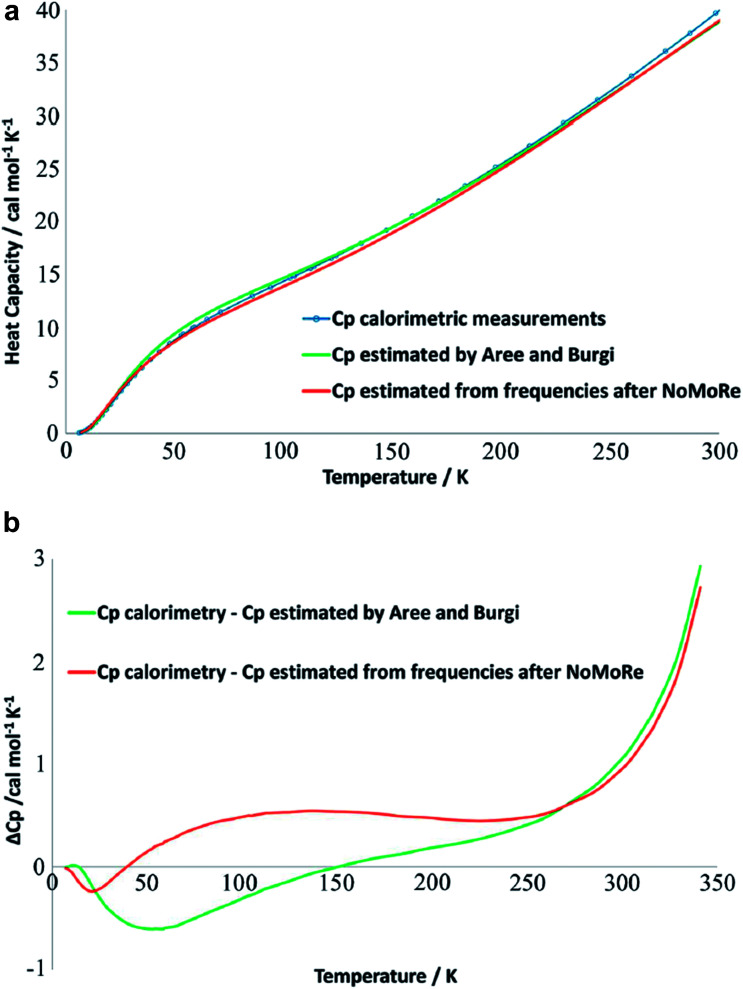
(a) Heat capacity (*C*_p_) for a molecular crystal of naphthalene: from thermodynamic measurements (blue curve), calculated from frequencies obtained after NoMoRe (red curve) and estimated by Aree and Bürgi from multi-temperature diffraction data^89^ (green curve). (b) Difference between *C*_p_ measured calorimetrically and *C*_p_ estimated with NoMoRe (red curve) or from multi-temperature data (green curve) (reprinted from ref. 90 under the general license agreement of IUCr journals).

This not only creates a need for simplified, yet accurate models, but also raises the question to what extent experimental mean square vibrational amplitudes (ADPs) can compensate for the simplifications. Hoser and Madsen have initiated what they call dynamic quantum crystallography.^86^ In this method, crystal normal modes and corresponding vibrational frequencies are determined from periodic *ab initio* calculations.^87^ The sampling of these modes is usually severely limited, often to the origin of the Brillouin zone (Γ-point). In the latter case the dynamical model encompasses only a single unit cell with *n* atoms and is thus highly oversimplified. The frequencies of the lowest crystal vibrations, the acoustic and low optical phonons, are particularly poorly represented in this approximation. These frequencies are therefore refined against experimental data until the ADPs derived from them, from the unmodified higher frequency phonons and from the refined atomic positions produce model structure factors *F*_model_ in agreement with X-ray or neutron diffraction data (Normal Mode Refinement, NoMoRe). Refined frequencies and calculated atomic displacement patterns compare well with results obtained independently from multi-temperature X-ray diffraction studies.^88^ Thermodynamic properties from NoMoRe and multi-temperature X-ray and neutron diffraction investigations^89^ agree – albeit not perfectly – with each other and with calorimetric data (Fig. 6).^90^ NoMoRe against X-ray data produces hydrogen atom ADPs that compare well with those from neutron diffraction data.

## 3. Quantum crystallography, second definition: enhancing experimental crystal structure models with information from quantum chemical calculations

### 3.1 Crystal structure determination by X-ray and electron diffraction has always used quantum-mechanical information

It is often forgotten that the standard models of crystal structure determination incorporate the experimentally determined structure factor amplitudes on one hand and atomic form factors calculated independently by *ab initio* methods on the other. Such models depend to a variable extent on the calculated information introduced into them. To illustrate this point, it is recalled that the measured X-ray diffraction intensities *I*_obs_(***h***) with Miller indices ***h*** = (*h*,*k*,*l*) are proportional to the square moduli of the structure factors |*F*_obs_(***h***)|:

7 *I*_obs_(***h***) ∼ |*F*_obs_(***h***)|^2^

The structure factors *F*(***h***), which are the Fourier transforms of the crystal unit-cell electron density,8
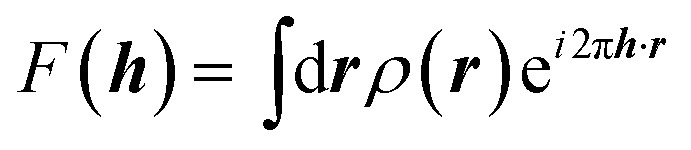
are modeled as a sum of atomic contributions9
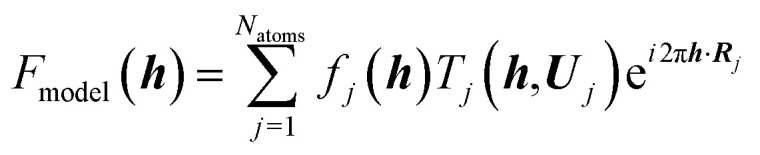
where *f*_*j*_ is the scattering or form factor of atom *j* at position ***R***_*j*_, and *T*_*j*_ is usually called ‘atomic temperature factor’, which depends on the mean square static and dynamic atomic displacements ***U***_*j*_ of atom *j* from its average position ***R***_*j*_. ***U***_*j*_ and ***R***_*j*_ are variables of *F*_model_ and are refined against the experimental data by minimizing the mean-square difference between |*F*_obs_(***h***)| and |*F*_model_(***h***)|. The atomic electron densities, *i.e.* the scattering factors *f*_*j*_ are not. From the beginning of X-ray crystal structure determination they have been taken as the Fourier transforms of calculated spherical electron densities of isolated atoms. In this model the *f*_*j*_ are assumed to always be the same, irrespective of an atom's chemical environment (Independent Atom Model, IAM).^91^ Early on, the atomic densities have been computed with the classical laws of electrodynamics,^92^ while later on they have been determined with increasingly sophisticated quantum mechanical models using, for example, Thomas–Fermi–Dirac^93^ or Hartree–Fock–Slater^94^ approximations.^95^ Most current software for crystal structure determination contains libraries with quantum-mechanical form factors calculated in the 1960s using relativistic wavefunctions.^96^ Reduced or increased scattering power of ions relative to the neutral atom is taken into consideration by Hartree–Fock calculations for lighter atoms,^97^ or relativistic Dirac–Slater calculations for heavier atoms.^98^

In contrast to X-ray diffraction, conventional electron diffraction experiments (for convergent-beam electron diffraction see Section 3.3) measure the interaction of the probing electrons with the electrostatic potential inside the crystal. Intensities *I*(***h***) are proportional to the square of the Fourier transform of the potential distribution *φ*(***r***). The latter can be modelled with isolated-atom scattering factors similar to eqn (9). Analytical Gaussian or polynomial fits to X-ray scattering factors are used to obtain numerical values of the electron-diffraction scattering factors.^96*a*,*b*,99^ Note that the nuclear scattering lengths and cross sections needed in neutron diffraction are obtained experimentally.

### 3.2 Aspherical scattering factors and electron-density research

The crystal field is never spherically symmetric, especially not if an atom forms strong directed bonds. Representing the crystal electron density as a sum of spherical atomic densities can thus be a severe approximation. Quantum-chemical calculations on molecules and polyatomic ions can account for distortions from spherical symmetry, but require procedures for partitioning the crystal electron density into a sum of atomic ones. The improved scattering factors are referred to as generalized or polarized-atom scattering factors. Stewart *et al.* suggested such scattering factors obtained from SCF calculations on diatomic and simple organic molecules.^100^ The treatment of the hydrogen atom is especially problematic, since hydrogen has no core electrons. The distribution of its single 1s-valence electron tends to be heavily deformed in covalent bonds. A scattering factor based on quantum-mechanical calculations of the H_2_ molecule has been especially successful for the treatment of such hydrogen atoms.^101^ A spherical approximation of the scattering factor of a hydrogen atom in the hydrogen molecule is still in use in most modern crystallographic software (Stewart–Davidson–Simpson, SDS, model).^102^

A different way to represent the crystal electron density in terms of aspherical atomic electron densities and their scattering factors is to parameterize pseudo-atom electron densities with analytical functions. The forms of the latter are based on quantum-chemical considerations, whereas their parameters are refined against the observed structure factors. Hence, such scattering factors are partly based on quantum mechanical theory and partly on diffraction data. Models in this family are known as multipole models. An incomplete list of the approaches that have been introduced and tested over the past 50 years includes the seminal work by Dawson 1967,^103^ Kurki-Suonio 1968,^104^ Stewart 1969,^81^ Hirshfeld 1971,^105^ Coppens 1971.^82^ The most widely used formalism is that of Hansen and Coppens from 1978.^106^ Its aspherical scattering factor expression is shown in eqn (10):10
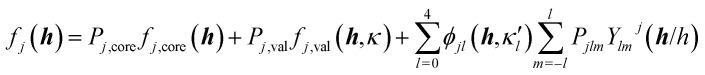


The core and valence spherical atomic scattering factors *f*_*j*,core_ and *f*_*j*,val_ are usually derived from Hartree–Fock wavefunctions expanded over Slater-type basis functions and tabulated in data-bases.^107^ Similarly the *ϕ*_*jl*_ functions are Fourier–Bessel transforms of radial density functions *R*_*jl*_ based on single-zeta Slater-type orbitals with energy-optimized exponents taken from valence-orbital wavefunction calculations.^107,108^*Y*_*lm*_^*j*^ are spherical harmonics. This means that the analytical form of the electron density is significantly influenced by quantum-chemical concepts and results, whereas the multipole population parameters *P*_*jlm*_, the expansion–contraction parameters *κ* and *κ*′ for the radial parts of the electron densities, positional parameters and ADPs are refined against the experimental diffraction data. In an effort to increase the flexibility of the multipole model, bonded-atom radial functions have been introduced.^109^ In an extended version of the Hansen–Coppens formalism, aspherical *f*_*j*,core_ account for core deformation and can be refined against the experimental data in exceptional cases.^110^

Atomic multipole descriptions derived from experiment and pertaining to different chemical environments have been collected in libraries of pseudoatoms (ELMAM).^111^ Multipole and expansion–contraction parameters have also been extracted from quantum-chemically computed electron densities of model compounds and tabulated for many atoms in a large but limited number of different chemical environments (invariom database,^112^ UBDB^113^). Using aspherical rather than spherical scattering factors in crystallographic refinements of positional parameters and ADPs improves the accuracy of the structural model.

### 3.3 Quantum crystallographic methods according to the second definition

As discussed above, crystal structure determination using the IAM or pseudo-atom models always involves quantum-mechanical information. Certain aspects of the crystal electron density are not measured, but modeled with the help of theory. From this viewpoint, every X-ray structure determined since the appearance of the Schrödinger equation in 1925 could be regarded as ‘quantum crystallography’. However, spherical atoms or pseudo-atoms in a standard chemical environment never reflect the influence of the actual chemical environment and the crystal field exactly. The quantum-chemical information introduced into the conventional structure modeling and refinement process is always approximate. The use of quantum chemistry in these methods may be considered as “indirect”, since it relies on tabulated information for model compounds.

In the spirit of Huang, Massa and Karle's definition of quantum crystallography in 1999,^2^ we consider quantum crystallography as an interdisciplinary approach that amalgamates numerical quantum chemical information into diffraction experiments within single, integrated tools. The use of the wavefunction is “direct” since it is an integral part of the refinement and modeling process, is tailor-made for the very molecular or extended system under investigation and is continuously updated during structure refinement.

**Fig. 7:**
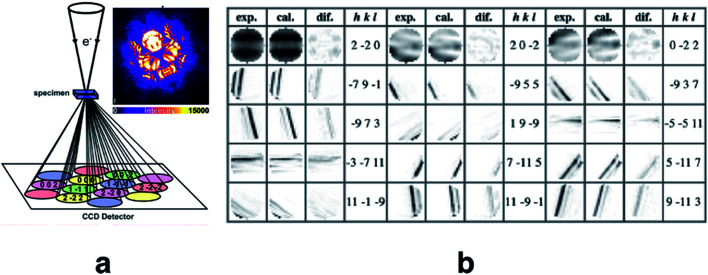
(a) Sketch of the scattering process leading to CBED disks that can be attributed to individual structure factors with Miller indices *hkl*. Intensity of the real measured pattern for aluminium in the crystal orientation 22̄0 is shown as an inlay. Reprinted from ref. 120 with permission from AAAS. (b) Final results of the fitting of the 22̄0 Bragg pattern of silicon giving rise to several very accurate structure factors from which the electron density distribution could be modeled. Reprinted from ref. 121 under the general license agreement of IUCr journals.

Quantitative convergent-beam electron diffraction (CBED) depends on such a dynamic model as it does not rely on tabulated atomic scattering factors. Each experiment requires its individual wavefunction to extract scattering amplitudes or Fourier potentials.^114^ To describe the multiple scattering between the incoming electron beam and the crystalline material a dynamical theory must be adopted. Dynamical Bloch-wave theory is often applied, which has some computational advantages for subsequent pattern matching procedures, and is similar to Pendellösung approaches for X-ray diffraction.^115^ The Schrödinger equation is solved for the single high-energy electron as it passes through the crystal and the electrostatic potential matrix elements are computed. In structure refinements from quantitative CBED data, simulated intensities are compared with observed intensity variations within CBED disks of different incident beam orientation (see Fig. 7). During refinement, wavefunction parameters in the dynamical theoretical model have to be updated repeatedly by adjustment of structural parameters and Debye–Waller factors.^116^ This “direct” combination of a diffraction experiment with theory can hence be considered the first quantum crystallographic technique according to the second definition. Method development and early applications date back to the 1960s.^117^ First interpretations of CBED disks with quantum chemical calculations appeared in the mid 1980s^118^ and were followed in the early 1990s by improved methods that take advantage of supercomputer facilities.^119^

**Fig. 8:**
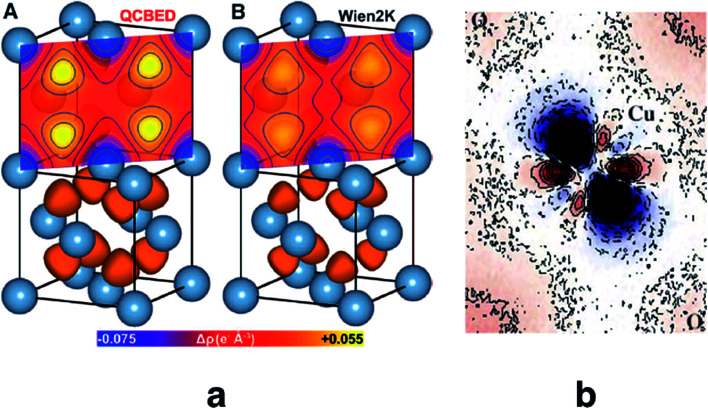
(a) Electron density of aluminium from quantitative CBED (left) and a periodic plane-wave calculation using the program Wien2k (right). Reprinted from ref. 120 with permission from AAAS. (b) Static deformation density map around a copper atom in Cu_2_O. Reprinted by permission from Macmillan Publishers Ltd.: Nature, ref. 122, copyright 1999.

Quantitative CBED leads to very accurate electron density distributions, but only for simple materials with few independent atoms in the unit cell. They are much more accurate than those based on X-ray diffraction because (i) scattering factors for electrons at low scattering angles are acutely sensitive to bonding, (ii) dynamic scattering effects are modelled, and (iii) the amount of data in a single CBED pattern provides orders of magnitude more data points than unknowns. Nakashima *et al.* demonstrated in 2011 that the bonding electron-density distribution of aluminium from quantitative CBED is more reliable than that from X-ray diffraction or theory (Fig. 8a) and that conclusions about anisotropic elastic constants can be drawn.^120^ Careful quantitative CBED experiments by Zuo *et al.* revealed bonding features between two copper atoms in Cu_2_O.^122^ The deformation electron density around the copper atoms (Fig. 8b) was interpreted in terms of a shift of electron density from a 3d orbital into a 4s orbital at the Cu atom. Static difference density maps (deformation density maps) between models based on aspherical and spherical atomic scattering factors (as discussed above) are not unusual in X-ray diffraction. However, the accuracy of the result from quantitative CBED is remarkable. Palatinus *et al.* have recently shown that hydrogen atoms can be located with precession electron diffraction tomography on nanocrystals if dynamical refinement is used.^123^ This finding complements the analogous capabilities of Hirshfeld atom refinement discussed below.

The first quantum crystallographic method in small-unit cell X-ray crystallography was introduced in 2008 by Jayatilaka and Dittrich.^124^ It is a two-step procedure called Hirshfeld Atom Refinement (HAR). In the first step, a molecular electron density is calculated from the atomic coordinates of an input geometry, typically at the Hartree–Fock or DFT level of approximation. The resulting electron density *ρ*_mol_ is partitioned into aspherical atomic electron densities *ρ*_*j*_ (Hirshfeld atoms) using the stockholder partitioning,^125^

11 *ρ*_*j*_(***r***) = *w*_*j*_(***r***)*ρ*_mol_(***r***)

with12
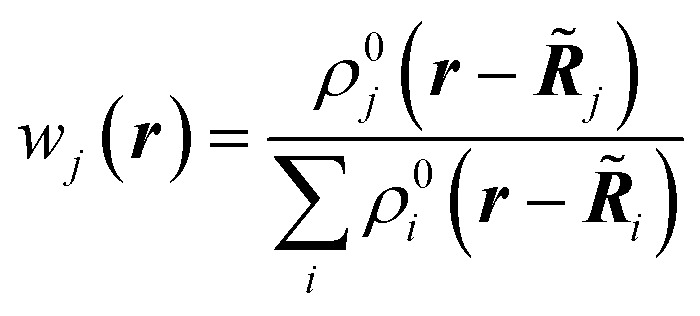
*w*_*j*_(***r***) is the weight function of atom *j* which carves an aspherical electron density out of the molecular electron density. It is defined as the ratio between the spherically averaged atomic electron density *ρ*^0^_j_(***r*** − ***R̃***_*j*_) of atom *j* at position ***R̃***_*j*_||||HAR operates in the Cartesian crystal coordinate system; it uses the scattering vector ***k*** = 2π***Bh*** (expressed in terms of the reciprocal cell matrix ***B***) and the positional atomic coordinates **R̃**_*j*_ rather than the dimensionless Miller indices ***h*** and the fractional coordinates ***R***_*j*_. and the sum of the spherical electron densities of all other atoms in the molecule. The spherically averaged atomic electron densities *ρ*^0^_j_(***r*** − ***R̃***_*j*_) are not taken from tables but calculated with the same quantum chemical model and basis-set as used for *ρ*_mol_. Optionally, atomic Hirshfeld charges and dipole moments can be obtained from the calculated molecular electron density and placed on atomic sites surrounding the central molecule in order to polarize the molecular electron density, thus simulating crystal field effects. In the field of these charges and dipoles a new molecular electron density and new charges and dipoles are calculated and the process iterated until self-consistency is reached. In preparation for the second step, namely the structural least-squares refinement, the static Hirshfeld atoms *ρ*_*j*_(***r***) are Fourier transformed to get the static, aspherical Hirshfeld scattering factors *f*_*j*_. They are included together with the usual temperature factor *T*_*j*_ for each atom *j* in the structure factor *F*_model_(***k***):13
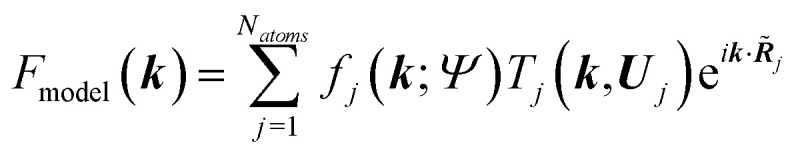
where *Ψ* stresses the dependence of the static Hirshfeld scattering factors on the calculated wavefunction. After refinement of the atomic positions ***R̃***_*j*_ and the atomic displacement parameters ***U***_*j*_, a new electron density is calculated with the updated atomic coordinates. In an automated and iterative version from 2014,^126^ the two-step process is repeated until the parameter shifts in ***R̃***_*j*_ and ***U***_*j*_ divided by their standard uncertainty are less than 0.01. A somewhat simplified version of HAR has recently become available in the standard Olex2 crystallographic software.^127^

The HAR procedure has advantages and disadvantages. On the one hand it ensures tailor-made aspherical atomic scattering factors. They optimally represent the unit-cell electron density within the limits of the chosen quantum mechanical procedure. This leads to enhanced crystal structure information in the spirit of the second definition of quantum crystallography. On the other hand the computational cost (CPU time) for a HAR refinement is significantly higher than for an IAM or multipole model refinement. Since the original HAR procedure is based on a molecular wavefunction, the treatment of periodic network structures is difficult. A recent variant of HAR based on periodic wavefunctions renders possible the treatment of network structures,^128^ and is potentially more accurate, but even more time-consuming.

**Fig. 9:**
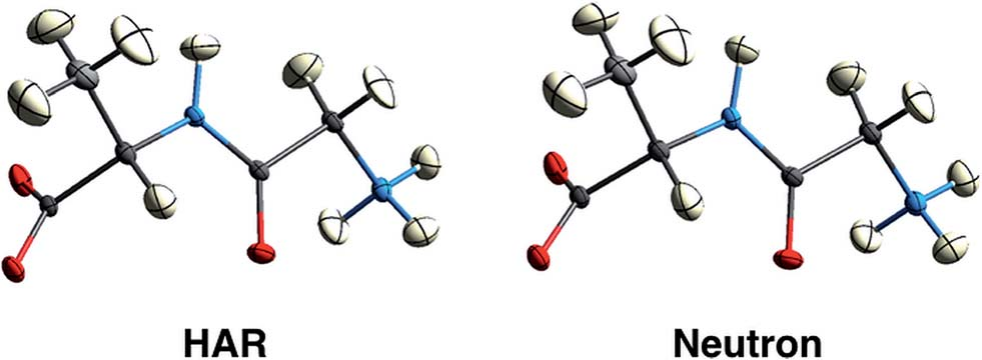
150 K structures of the dipeptide Gly-*L*-Ala with anisotropic displacement parameters (ADPs) at 50% probability from HAR based on X-ray diffraction data (left) and from neutron diffraction data (right). The structures are virtually identical by visual inspection. Reprinted from ref. 126 under the general license agreement of IUCr journals.

First results obtained with HAR procedures are encouraging in two ways. Firstly, as Wall points out:^128^ “the results indicate that HAR can yield not only molecular geometries and ADPs that are similar to the neutron crystal structure, but also both 2Fo–Fc maps and static charge densities that are distinct from the multipole model, but that nevertheless agree comparably with the experimental data. Quantum crystallography therefore can yield accurate charge densities that are consistent simultaneously with theory and experiment”. Chęcińska *et al.*^129^ and Dittrich *et al.*^130^ have reached similar conclusions in earlier electron-density research involving HAR. Secondly, several groups have shown that for data sets of good quality, hydrogen atomic positions and ADPs can be freely refined and are generally found in quantitative agreement with results from neutron diffraction,^126,131,132^ see Fig. 9 and Table 1. This holds for a large range of organic molecules and for data sets with resolutions as low as 0.8 Å, which can routinely be measured at in-house diffractometers with conventional X-radiation sources.^131^ Studies in inorganic chemistry are currently ongoing.

**Table 1:** Mean absolute differences and their population standard deviations for the dipeptide Gly-*L*-Ala in terms of bond distances *d* involving hydrogen atoms and ADPs ***U***_*ij*_ of hydrogen atoms between HAR (based on X-ray diffraction) and neutron diffraction results for four different temperatures^126^

*T* in K	〈|Δ*d*|〉 in Å	〈|Δ***U***_*ij*_|〉 in Å^2^
12	0.0085(51)	0.0047(42)
50	0.0093(52)	0.0040(34)
150	0.0087(62)	0.0037(31)
295	0.0125(84)	0.0075(58)

### 3.4 Quantum crystallography in protein structure determination

Macromolecular crystallographic refinement is another area in which quantum mechanical calculations can supplement the information provided by X-ray diffraction measurements. As is well known, one of the main obstacles preventing atomic-level resolutions of macromolecular crystallographic structures is the limited resolution and number of diffraction data compared to the number of parameters needed to model atomic positions and thermal motion. To overcome this drawback, constraints and restraints^133^ are usually introduced in the refinement. While the former reduce the number of structural parameters to be determined, the latter introduce stereochemical information into the analysis that complements the one provided by the X-ray data. Restraints take the form of penalty terms dependent on the deviation from reference parameters – similar to energy penalties in molecular-mechanics (MM) force fields, although with some differences: (i) the reference parameters are generally obtained from a statistical analysis of a large number of accurate protein and polynucleotide crystallographic structures, as done by Engh & Huber^134^ who exploited the Cambridge Structural Database (CSD); (ii) electrostatic interactions and van der Waals attractions are neglected.^135^ With such tailor-made restraints, accurate structures of proteins have been determined. However, when a macromolecule harbours a small molecule, *e.g.* a ligand, an inhibitor, or a cofactor with or without a metal atom, accurate restraint parameters for the molecule are often not available and an accurate structural model is difficult to achieve.

To alleviate this problem, Ryde and coworkers developed the ComQum-X method. It combines information from multi-scale Quantum Mechanical/Molecular Mechanical (QM/MM) calculations with that from experimental structure factors.^136^ The following functional is minimized:

14 *E*_ComQum−*X*_ = *E*_QM/MM_ + *ω*_*X*−ray_*E*_*X*−ray_.

*E*_QM/MM_ is the QM/MM energy of the investigated system; *E*_*X*−ray_ is the crystallographic penalty function, which measures the agreement between the observed and the calculated structure factors; *ω*_*X*−ray_ is a weight that balances the computational and the experimental information. The QM part encompasses the active site of proteins, their ligands and the nearby residues. The rest of the system is treated at the molecular mechanics level. The QM/MM calculation for the entire system is part of each step of the refinement, in agreement with the quantum crystallography idea. Crystallographic structures of several proteins containing ligands have been refined with the ComQum-X technique.^136,137^

**Fig. 10:**
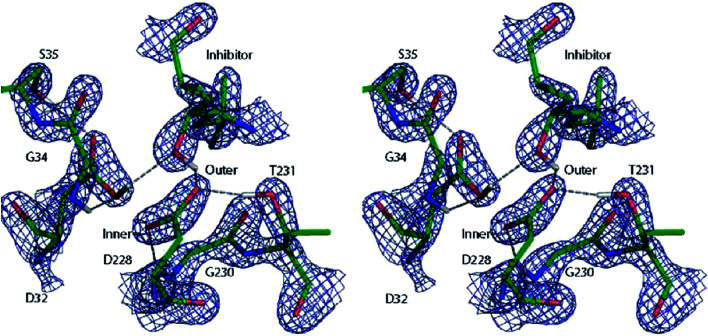
Cross-eye stereo view of the 2*F*_o_ − *F*_c_ electron density map (2.7*σ* contour level) around the active site residues of the β-secretase enzyme after QM/MM refinement of the most favoured 32i state. Note the hydrogen bond formed between the hydroxyl group of the inhibitor and the carboxyl group of residue D32. Reprinted with permission from ref. 138a. Copyright 2006 American Chemical Society.

A similar QM/MM approach was implemented and applied by Merz and coworkers.^138^ They successfully assigned protonation states of key residues in β-secretase, crucial in the pathogenesis of Alzheimer's disease (see Fig. 10),^138*a*^ and reliably described the zinc coordination in zinc metalloenzymes.^138*c*^

The Merz group also proposed to replace the QM/MM computations of the ComQum-X approach by semiempirical quantum mechanical calculations.^139^ In order to be able to treat large molecular systems, they exploited their linear scaling “Divide & Conquer” (D&C) semiempirical method^140^ implemented in the program DivCon. The functional to be minimized now reads:

15 *E*_DivCon−*X*_ = *E*_D&C_ + *ω*_*X*−ray_*E*_*X*−ray_

*E*_D&C_ is the semiempirical quantum mechanical energy of the system from the “Divide & Conquer” approach. As in the QM/MM example, the D&C calculations are performed at each step of the refinement.

**Fig. 11:**
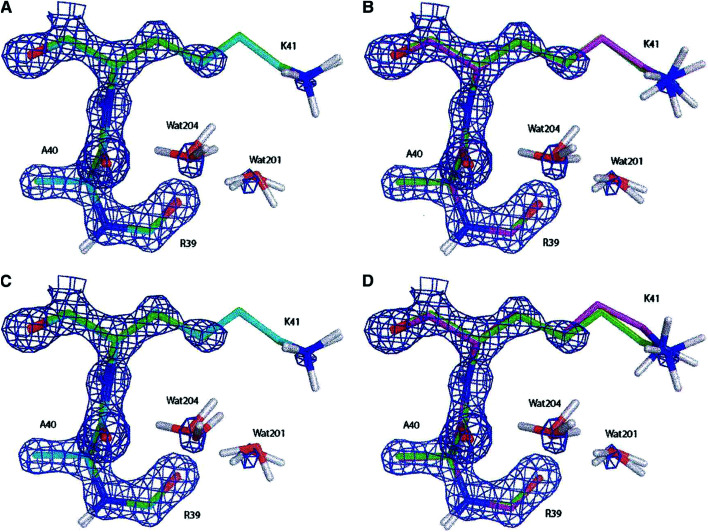
Electron density maps of residues Ala40, Lys41 and Arg30 (and two water molecules) of the bovine pancreatic trypsin inhibitor (2.3*σ* contour level) after (A) a full QM refinement with *ω*_*X*−ray_ = 0.9 (cyan), (B) an EH-restrained refinement with *ω*_*X*−ray_ = 0.9 (magenta), (C) a full QM refinement with *ω*_*X*−ray_ = 0.2 (cyan), (D) an EH-restrained refinement with *ω*_*X*−ray_ = 0.2 (magenta). All the obtained structures are superimposed to the 5PTI structure (green). Note the differences in hydrogen atom positions (aliphatic hydrogen atoms omitted for clarity). Reprinted from ref. 139b with the permission of WILEY.

The method was tested by refining the crystallographic structure of the bovine pancreatic trypsin inhibitor. The QM refined structure and the one resulting from an Engh & Huber (EH) refinement were compared to the structure obtained from a conventional joint refinement of X-ray and neutron diffraction data not applying any restraints (Protein Databank (PDB) code 5PTI).^139^ The “Divide & Conquer” strategy provided improvements, mainly for hydrogen atom coordinates compared to the approach based on the EH restraints (see Fig. 11). The main reason for this improvement is the direct inclusion in the fully quantum mechanical refinement of electrostatics which is completely missing in the EH-based technique. The D&C strategy is now included^141^ in the popular crystallographic package PHENIX.^142^ The new, slightly different, algorithm was tested against a set of fifty protein-ligand structures obtained from the PDB.^141^ It always provided completely plausible ligand geometries and low ligand strains, also in cases in which the original, traditional refinement showed some difficulties.

## 4. Conclusion and outlook

Quantum crystallography encompasses two streams of research both of which rely on an integrated and self-consistent combination of quantum chemistry and crystallographic information. One of them pertains to the enhancement of quantum chemical information by fitting wavefunction parameters against diffraction and scattering data in an integrated way. In the second stream, the information from crystallographic diffraction experiments is enhanced and its accuracy and precision improved by the integrated use of quantum-chemical data in the modeling process. For the two streams, the use of the term ‘integrated’ implies (i) adjusting calculated wavefunction parameters to be consistent with experimentally determined diffraction and/or scattering data (first definition), or (ii) iteratively adjusting atomic positions and displacement parameters with the help of quantum-chemically recalculated electron densities (second definition). The latter procedure does not rely on pre-calculated and tabulated spherical or aspherical scattering factors as have been used in traditional crystal structure determination and electron-density research. Some of the new techniques are now part of standard crystallographic software packages such as Olex2 or PHENIX.

Quantum crystallography is an active research field, whose potential has not been fully exploited yet. New methods and results appear every year. Here we have presented examples for

• Modeling of accurate electron-density distributions for bonding analysis in simple materials (quantitative convergent beam electron diffraction);

• Determination of hydrogen atom positions and hydrogen ADPs in small molecules from X-ray diffraction experiments with an accuracy comparable to that obtainable from the more involved neutron diffraction experiments (Hirshfeld Atom Refinement, HAR);

• Improved structure determination in protein crystallography providing an extra level of insight into modes of action of biological macromolecules (QM/MM and Divide & Conquer approaches);

• Characterization of bonding and recovering traditional Lewis pictures of electronic structure (XC-ELMO valence bond and X-ray constrained localization functions);

• Development of computational strategies aiming at quantum crystallographic wavefunctions, which account for electron correlation, polarization and relativistic effects, and may be viewed as complements to those calculated with *ab initio* and DFT methods;

• X-ray wavefunction refinement (XWR), a sequential combination of HAR and X-ray constrained wavefunction (XCW) fitting:^80,143^ HAR leads to improved structural parameters, and subsequent XCW fitting yields an improved and enhanced quantum-chemical wavefunction. Hence, XWR is the only quantum crystallographic technique conforming to both aspects of the definition.

• Determination of physical properties of materials such as dipole moments, polarizabilities, hyperpolarizabilities, or refractive indices from XCWs;

• Determination of crystal vibrational frequencies from a combination of periodic lattice dynamical *ab initio* calculations and measured diffraction intensities (dynamic quantum crystallography).

We believe that in the near future quantum crystallographic methods will become available for

• Developing or at least improving DFT functionals with the help of experimentally determined electron correlation effects;

• Computing accurate cohesive energies from XCWs and specific heat curves from normal mode refinements with the aim of predicting polymorphs more reliably;

• Refining crystallographic structures and reconstructing electron densities of large macromolecules, especially if the novel libraries of (XC-)ELMOs,^144^ which are alternatives to the widely used pseudoatoms databanks of the multipole models, will be combined with HAR;

• Dynamic updating of geometry, ADPs and wavefunction within a single procedure. This can be achieved through automated XWR, so that both quantum crystallographic structure refinement and wavefunction fitting are carried out iteratively or even simultaneously until convergence in changes of wavefunction and structural parameters are obtained.

We believe that the methods of quantum crystallography reviewed here represent – already today – a useful set of tools for research in chemical bonding, crystal dynamics and quantum chemistry.

## Supplementary Material

## References

[cit1] Massa L., Huang L., Karle J. (1995). Int. J. Quantum Chem., Quantum Chem. Symp..

[cit2] Huang L., Massa L., Karle J. (1999). Int. J. Quantum Chem..

[cit3] Macchi P., Gillet J.-M., Taulelle F., Campo J., Claiser N., Lecomte C. (2015). IUCrJ.

[cit4] Mukherji A., Karplus M. (1963). J. Chem. Phys..

[cit5] Rasiel Y., Whitman D. R. (1965). J. Chem. Phys..

[cit6] Byers Brown W. (1966). J. Chem. Phys..

[cit7] Fraga S., Birss F. W. (1966). Theor. Chim. Acta.

[cit8] Clinton W. L., Nakhleh J., Wunderlich F. (1969). Phys. Rev..

[cit9] Clinton W. L., Galli A. J., Massa L. J. (1969). Phys. Rev..

[cit10] Clinton W. L., Lamers G. B. (1969). Phys. Rev..

[cit11] Clinton W. L., Galli A. J., Henderson G. A., Lamers G. B., Massa L. J., Zarur J. (1969). Phys. Rev..

[cit12] Löwdin P. O. (1955). Phys. Rev..

[cit13] McWeeny R. (1960). Rev. Mod. Phys..

[cit14] Coleman A. J. (1963). Rev. Mod. Phys..

[cit15] Clinton W. L., Massa L. J. (1972). Int. J. Quantum Chem..

[cit16] Clinton W. L., Massa L. J. (1972). Phys. Rev. Lett..

[cit17] Clinton W. L., Frishberg C. A., Massa L. J., Oldfield P. A. (1973). Int. J. Quantum Chem., Quantum Chem. Symp..

[cit18] Frishberg C. A., Massa L. J. (1978). Int. J. Quantum Chem..

[cit19] Frishberg C., Massa L. J. (1981). Phys. Rev. B: Condens. Matter Mater. Phys..

[cit20] Frishberg C. A., Massa L. J. (1982). Acta Crystallogr., Sect. A: Cryst. Phys., Diffr., Theor. Gen. Crystallogr..

[cit21] Boehme R. F., La Placa S. J. (1987). Phys. Rev. Lett..

[cit22] Frishberg C. (1986). Int. J. Quantum Chem..

[cit23] Clementi E. (1965). IBM J. Res. Dev..

[cit24] Kutzelnigg W., Smith Jr V. H. (1964). J. Chem. Phys..

[cit25] Benesch R., Smith Jr V. H. (1970). Acta Crystallogr., Sect. A: Cryst. Phys., Diffr., Theor. Gen. Crystallogr..

[cit26] Barnett G., Linderberg J., Shull H. (1965). J. Chem. Phys..

[cit27] Massa L., Goldberg M., Frishberg C., Boehme R. F., La Placa S. J. (1985). Phys. Rev. Lett..

[cit28] Aleksandrov Y. V., Tsirelson V. G., Reznik I. M., Ozerov R. P. (1989). Phys. Status Solidi B.

[cit29] Goldberg M. J., Massa L. J. (1983). Int. J. Quantum Chem..

[cit30] Pecora L. M. (1986). Phys. Rev. B: Condens. Matter Mater. Phys..

[cit31] Howard S. T., Huke J. P., Mallinson P. R., Frampton C. S. (1994). Phys. Rev. B: Condens. Matter Mater. Phys..

[cit32] Snyder J. A., Stevens E. D. (1999). Chem. Phys. Lett..

[cit33] Figgis B. N., Kucharski E. S., Williams G. A. (1980). J. Chem. Soc., Dalton Trans..

[cit34] Tanaka K. (1988). Acta Crystallogr., Sect. A: Found. Crystallogr..

[cit35] Tanaka K. (1993). Acta Crystallogr., Sect. B: Struct. Sci..

[cit36] Tanaka K., Kato Y., Onuki Y. (1997). Acta Crystallogr., Sect. B: Struct. Sci..

[cit37] Tanaka K., Onuki Y. (2002). Acta Crystallogr., Sect. B: Struct. Sci..

[cit38] Tanaka K., Makita R., Funahashi S., Komori T., Win Z. (2008). Acta Crystallogr., Sect. A: Found. Crystallogr..

[cit39] Hibbs D. E., Howard S. T., Huke J. P., Waller M. P. (2005). Phys. Chem. Chem. Phys..

[cit40] Schmider H., Smith Jr V. H., Weyrich W. (1990). Trans. Am. Crystallogr. Assoc..

[cit41] Gillet J.-M., Becker P. J. (2004). J. Phys. Chem. Solids.

[cit42] Nicholson R. J. F., McCarthy I. E., Brunger M. J. (1998). Aust. J. Phys..

[cit43] Cassam-Chenaï P. (1995). Int. J. Quantum Chem..

[cit44] Cassam-Chenaï P., Wolff S., Chandler G., Figgis B. (1996). Int. J. Quantum Chem..

[cit45] Gilbert T. L. (1975). Phys. Rev. B: Condens. Matter Mater. Phys..

[cit46] Henderson G. A., Zimmermann R. K. (1976). J. Chem. Phys..

[cit47] Kryachko E. S., Petkov I. Z., Stoitsov M. (1987). Int. J. Quantum Chem..

[cit48] Petkov I. Z., Stoitsov M., Kryachko E. S. (1986). Int. J. Quantum Chem..

[cit49] Levy M., Goldstein J. A. (1987). Phys. Rev. B: Condens. Matter Mater. Phys..

[cit50] Gritsenko O. V., Zhidomirov G. M. (1987). Dokl. Akad. Nauk SSSR.

[cit51] Zhao Q., Parr R. G. (1992). Phys. Rev. A.

[cit52] Nyden M. R., Parr R. G. (1983). J. Chem. Phys..

[cit53] Dawson K. A., March N. H. (1984). J. Chem. Phys..

[cit54] Jayatilaka D. (1998). Phys. Rev. Lett..

[cit55] Jayatilaka D., Grimwood D. J. (2001). Acta Crystallogr., Sect. A: Found. Crystallogr..

[cit56] Grimwood D. J., Jayatilaka D. (2001). Acta Crystallogr., Sect. A: Found. Crystallogr..

[cit57] Bytheway I., Grimwood D. J., Jayatilaka D. (2002). Acta Crystallogr., Sect. A: Found. Crystallogr..

[cit58] Bytheway I., Grimwood D. J., Figgis B. N., Chandler G. S., Jayatilaka D. (2002). Acta Crystallogr., Sect. A: Found. Crystallogr..

[cit59] Grimwood D. J., Bytheway I., Jayatilaka D. (2003). J. Comput. Chem..

[cit60] Jayatilaka D. , in Modern Charge-Density Analysis, ed. C. Gatti and P. Macchi, Springer, Berlin, 2012, vol. 6, pp. 213–257

[cit61] Levy M. (1979). Proc. Natl. Acad. Sci. U. S. A..

[cit62] Hudák M., Jayatilaka D., Perašínova L., Biskupic S., Kozísek J., Bučinský L. (2010). Acta Crystallogr., Sect. A: Found. Crystallogr..

[cit63] Bučinský L., Jayatilaka D., Grabowsky S. (2016). J. Phys. Chem. A.

[cit64] Stoll H., Wagenblast G., Preuss H. (1980). Theor. Chim. Acta.

[cit65] Sironi M., Genoni A., Civera M., Pieraccini S., Ghitti M. (2007). Theor. Chem. Acc..

[cit66] Genoni A. (2013). J. Phys. Chem. Lett..

[cit67] Genoni A. (2013). J. Chem. Theory Comput..

[cit68] Dos Santos L. H. R., Genoni A., Macchi P. (2014). Acta Crystallogr., Sect. A: Found. Adv..

[cit69] Genoni A., Meyer B. (2016). Adv. Quantum Chem..

[cit70] Whitten A. E., Jayatilaka D., Spackman M. (2006). J. Chem. Phys..

[cit71] Jayatilaka D., Munshi P., Turner M. J., Howard J. A. K., Spackman M. A. (2009). Phys. Chem. Chem. Phys..

[cit72] Hickstein D. D., Cole J. M., Turner M. J., Jayatilaka D. (2013). J. Chem. Phys..

[cit73] Cole J. M., Hickstein D. D. (2013). Phys. Rev. B: Condens. Matter Mater. Phys..

[cit74] Becke A. D., Edgecombe K. E. (1990). J. Chem. Phys..

[cit75] Kohout M. (2004). Int. J. Quantum Chem..

[cit76] Bader R. F. W., Stephens M. E. (1975). J. Am. Chem. Soc..

[cit77] Jayatilaka D., Grimwood D. J. (2004). Acta Crystallogr., Sect. A: Found. Crystallogr..

[cit78] Grabowsky S., Jayatilaka D., Mebs S., Luger P. (2010). Chem.–Eur. J..

[cit79] Grabowsky S., Weber M., Jayatilaka D., Chen Y. S., Grabowski M. T., Brehme R., Hesse M., Schirmeister T., Luger P. (2011). J. Phys. Chem. A.

[cit80] Grabowsky S., Luger P., Buschmann J., Schneider T., Schirmeister T., Sobolev A. N., Jayatilaka D. (2012). Angew. Chem., Int. Ed..

[cit81] Stewart R. F. (1969). J. Chem. Phys..

[cit82] Coppens P., Willoughby T. V., Csonka L. N. (1971). Acta Crystallogr., Sect. A: Cryst. Phys., Diffr., Theor. Gen. Crystallogr..

[cit83] Genoni A., Dos Santos L. H. R., Meyer B., Macchi P. (2017). IUCrJ.

[cit84] Genoni A. (2016). Acta Crystallogr., Sect. A: Found. Adv..

[cit85] Casati N., Kleppe A., Jephcoat A. P., Macchi P. (2016). Nat. Commun..

[cit86] Hoser A. A., Madsen A. Ø. (2016). Acta Crystallogr., Sect. A: Found. Adv..

[cit87] Madsen A. Ø., Civalleri B., Ferrabone M., Pascale F., Erba A. (2013). Acta Crystallogr., Sect. A: Found. Crystallogr..

[cit88] Bürgi H.-B., Capelli S. C. (1995). Acta Crystallogr., Sect. A: Found. Crystallogr..

[cit89] Aree T., Bürgi H.-B. (2006). J. Phys. Chem. B.

[cit90] Hoser A. A., Madsen A. Ø. (2017). Acta Crystallogr., Sect. A: Found. Adv..

[cit91] Compton A. H. (1915). Nature.

[cit92] James R. W., Brindley G. W. (1931). Philos. Mag..

[cit93] Abrahamson A. A. (1961). Phys. Rev..

[cit94] Hanson H. P., Herman F., Lea J. D., Skillman S. (1964). Acta Crystallogr..

[cit95] Cromer D. T. (1965). Acta Crystallogr..

[cit96] (c) Brown P. J. , Fox A. G., Maslen E. N., O'Keefe M. A. and Willis B. T. M., in International Tables For Crystallography, Volume C, ed. E. Prince, Wiley, Chichester, U.K., 3rd edn, 1999, vol. 6.1, pp. 554–595

[cit97] Cromer D. T. and Mann J. B., Los Alamos Scientific Laboratory Report LA-3816, 1968

[cit98] Cromer D. T. and Waber J. T., 1968, as reported in International Tables For Crystallography, Kynoch Press, 1974, vol. IV, p. 71

[cit99] Rez D., Rez P., Grant I. (1994). Acta Crystallogr., Sect. A: Found. Crystallogr..

[cit100] Bentley J., Stewart R. F. (1973). J. Comput. Phys..

[cit101] Destro R., Marsh R. E., Bianchi R. (1988). J. Phys. Chem..

[cit102] Stewart R. F., Davidson E. R., Simpson W. T. (1965). J. Chem. Phys..

[cit103] Dawson B. (1967). Proc. R. Soc. London, Ser. A.

[cit104] Kurki-Suonio K. (1968). Acta Crystallogr., Sect. A: Cryst. Phys., Diffr., Theor. Gen. Crystallogr..

[cit105] Hirshfeld F. L. (1971). Acta Crystallogr., Sect. B: Struct. Crystallogr. Cryst. Chem..

[cit106] Hansen N. K., Coppens P. (1978). Acta Crystallogr., Sect. A: Cryst. Phys., Diffr., Theor. Gen. Crystallogr..

[cit107] Clementi E., Roetti C. (1974). At. Data Nucl. Data Tables.

[cit108] Clementi E., Raimondi D. L. (1963). J. Chem. Phys..

[cit109] Koritsanszky T., Volkov A. (2004). Chem. Phys. Lett..

[cit110] Fischer A., Tiana D., Scherer W., Batke K., Eickerling G., Svendsen H., Bindzus N., Iversen B. B. (2011). J. Phys. Chem. A.

[cit111] Domagała S., Fournier B., Liebschner D., Guillot B., Jelsch C. (2012). Acta Crystallogr., Sect. A: Found. Crystallogr..

[cit112] Dittrich B., Hübschle C. B., Pröpper K., Dietrich F., Stolper T., Holstein J. J. (2013). Acta Crystallogr., Sect. B: Struct. Sci., Cryst. Eng. Mater..

[cit113] Jarzembska K. N., Dominiak P. M. (2012). Acta Crystallogr., Sect. A: Found. Crystallogr..

[cit114] Spence J. C. H. (1993). Acta Crystallogr., Sect. A: Found. Crystallogr..

[cit115] Hirsch P. , Howie A., Nicholson R. B., Pashley D. W. and Whelan M. J., Electron Microscopy of Thin Crystals, Krieger, Florida, U.S.A., 1977

[cit116] Zuo J. M. , in Electron Crystallography: Novel Approaches for Structure Determination of Nanosized Materials, Volume 211 of the NATO Science Series: II. Mathematics, Physics and Chemistry, ed. T. E. Weirich, J. L. Lábár and X. Zou, Springer, Dordrecht, The Netherlands, 2006, B.6, pp. 143–168

[cit117] Cockayne D. J. H., Goodman P., Mills J. C., Moodie A. F. (1967). Rev. Sci. Instrum..

[cit118] Taftø T., Metzger T. H. (1985). J. Appl. Crystallogr..

[cit119] Bird D. M., Saunders M. (1992). Acta Crystallogr., Sect. A: Found. Crystallogr..

[cit120] Nakashima P. N. H., Smith A. E., Etheridge J., Muddle B. C. (2011). Science.

[cit121] Ogata Y., Tsuda K., Tanaka M. (2008). Acta Crystallogr., Sect. A: Found. Crystallogr..

[cit122] Zuo J. M., Kim M., O'Keeffe M., Spence J. C. H. (1999). Nature.

[cit123] Palatinus L., Brázda P., Boullay P., Perez O., Klementová M., Petit S., Eigner V., Zaarour M., Mintova S. (2017). Science.

[cit124] Jayatilaka D., Dittrich B. (2008). Acta Crystallogr., Sect. A: Found. Crystallogr..

[cit125] Hirshfeld F. L. (1977). Isr. J. Chem..

[cit126] Capelli S. C., Bürgi H.-B., Dittrich B., Grabowsky S., Jayatilaka D. (2014). IUCrJ.

[cit127] (b) Fugel M. , Jayatilaka D., Hupf E., Turner M. J., Overgaard J., Hathwar V. R., Iversen B. B., Macchi P., Bürgi H.-B., Howard J. A. K., Dolomanov O. V., Puschmann H. and Grabowsky S., manuscript in preparation

[cit128] Wall M. E. (2016). IUCrJ.

[cit129] Chęcińska L., Morgenroth W., Paulmann C., Jayatilaka D., Dittrich B. (2013). CrystEngComm.

[cit130] Dittrich B., Sze E., Holstein J. J., Hübschle C. B., Jayatilaka D. (2012). Acta Crystallogr., Sect. A: Found. Crystallogr..

[cit131] Woińska M., Jayatilaka D., Spackman M. A., Edwards A. J., Dominiak P. M., Woźniak K., Nishibori E., Sugimoto K., Grabowsky S. (2014). Acta Crystallogr., Sect. A: Found. Adv..

[cit132] Dittrich B., Lübben J., Mebs S., Wagner A., Luger P., Flaig R. (2017). Chem.–Eur. J..

[cit133] Jack A., Levitt M. (1978). Acta Crystallogr., Sect. A: Cryst. Phys., Diffr., Theor. Gen. Crystallogr..

[cit134] Engh R. A., Huber R. (1991). Acta Crystallogr., Sect. A: Found. Crystallogr..

[cit135] Brünger A. T., Adams P. D. (2002). Acc. Chem. Res..

[cit136] Ryde U., Olsen L., Nilsson K. (2002). J. Comput. Chem..

[cit137] Ryde U., Nilsson K. (2003). J. Am. Chem. Soc..

[cit138] Yu N., Hayik S. A., Wang B., Liao N., Reynolds C. H., Merz Jr K. M. (2006). J. Chem. Theory Comput..

[cit139] Yu N., Yennawar H. P., Merz Jr K. M. (2005). Acta Crystallogr., Sect. D: Biol. Crystallogr..

[cit140] Dixon S. L., Merz Jr K. M. (1996). J. Chem. Phys..

[cit141] Borbulevych O. Y., Plumley J. A., Martin R. I., Merz Jr K. M., Westerhoff L. M. (2014). Acta Crystallogr., Sect. D: Biol. Crystallogr..

[cit142] Adams P. D. (2010). Acta Crystallogr., Sect. D: Biol. Crystallogr..

[cit143] (b) Woińska M. , Jayatilaka D., Dittrich B., Flaig R., Luger P., Wozniak K., Dominiak P. M. and Grabowsky S., manuscript in preparation 10.1002/cphc.201700810 29168318

[cit144] Meyer B., Guillot B., Ruiz-Lopez M. F., Genoni A. (2016). J. Chem. Theory Comput..

